# Analytical Model of ALOHA and Time- and Frequency-Asynchronous ALOHA with Forward Error Correction for IoT Systems

**DOI:** 10.3390/s22103741

**Published:** 2022-05-14

**Authors:** Federico Clazzer, Marcel Grec

**Affiliations:** German Aeropsace Center (DLR), Institute of Communications and Navigation, 82234 Weßling, Germany; marcel.grec@dlr.de

**Keywords:** machine-type communications, grant-free access, IoT via satellite

## Abstract

The blooming of internet of things (IoT) services calls for a paradigm shift in the design of communications systems. Short data packets sporadically transmitted by a multitude of low-cost low-power terminals require a radical change in relevant aspects of the protocol stack. For example, scheduling-based approaches may become inefficient at the medium access (MAC) layer, and alternatives such as uncoordinated access policies may be preferred. In this context random access (RA) in its simplest form, i.e., additive links on-line Hawaii area (ALOHA), may again become attractive as also proved by a number of technologies adopting it. The use of forward error correction (FEC) can improve its performance, yet a comprehensive analytical model including this aspect is still missing. In this paper, we provide a first attempt by deriving exact expressions for the packet loss rate and spectral efficiency of ALOHA with FEC, and extend the result also to time- and frequency-asynchronous ALOHA aided by FEC. We complement our study with extensive evaluations of the expressions for relevant cases of study, including an IoT system served by low-Earth orbit (LEO) satellites. Non-trivial outcomes show how time- and frequency-asynchronous ALOHA particularly benefit from the presence of FEC and become competitive with ALOHA.

## 1. Introduction

The broad topic of internet of things (IoT) communications is increasingly gathering industrial and research interest. Not only is IoT traffic predicted to become predominant in future wireless systems, but also a blooming number of disruptive application areas are posing accents on different and sometimes opposing performance metrics: while classical communications deal with the spectrum-sharing of few data-rate hungry terminals that typically have predictable resource requirements, the IoT traffic shifts the paradigm of communications. A large population of, sometimes, low-cost and low-power devices need to share the spectrum while sending small data packets in a very sporadic and unpredictable or event-driven way. Emerging use cases relying totally or in part on IoT traffic range from smart agriculture [1] to industry 4.0 [2], from environmental monitoring [3] to logistics [4] and the smart city [5]. In the first area, the possibility to automatically monitor both animals and plants can improve their well-being, as well as increase the production or harvest. The installation of smart sensors and actuators inside the factory of the future allows the automatic monitoring of goods production. Robots and automated machines can be interconnected so as to enhance their capabilities and allow complex tasks. Not only autonomous driving but also the support of remote monitoring will revolutionize logistics, enabling for example real-time status checks on the moving vehicles as well as automatic anomaly detection. The deployment of smart sensors in the environment opens up to a plurality of services. Alerting systems may be used to identify fire outbreaks, flooding, earthquakes, and all other types of disasters. The possibility to attach smart sensors to every infrastructure in the smart city, enables us to bring city monitoring to a whole new level. Infrastructures including the energy, water, gas and heating supply networks could be automatically monitored. Not only that, but also mobile communication infrastructure, roads, security systems, buildings can all be integrated and monitored.

The paradigm shift posed by IoT traffic has a deep impact on the entire protocol stack and especially the physical and medium access (MAC) layers require dedicated tailoring. In particular, short packet transmission calls for efficient short codes able to counteract the channel effects and multi-user interference and that are amenable to easing channel estimation [6]. In fact, the use of data-aided channel estimation may result in an unacceptable waste of resources, especially when the information to be sent is very small, for example as low as 100 bits. Traditional scheduling-based medium access methods become inefficient when sporadic transmission of short packets takes place. The overhead to allocate—possibly dynamically—the resources for a vast population of terminals where only a small and variable fraction is active at a time, may become unacceptably large and can even overcome the resources needed for data transmission.

In this context, random access (RA) protocols emerge as valid alternatives thanks to their flexibility regarding the number of supported users, and dispensable allocation of pre-defined resources [7]. Uncoordinated transmissions have the benefit of flexibility and require no scheduling overhead at the expense of rising multi-user interference. In its simplest form, also known as the additive links on-line Hawaii area (ALOHA) protocol [8], nodes are permitted to send their message immediately upon generation, regardless of the channel activity. If packets are involved in a collision, randomized retransmissions are utilized to resolve contention. The first extension to ALOHA has been proposed few years later under the name of slotted ALOHA (SA) [9]. The simple yet brilliant idea to let users access the medium only in a slotted fashion drastically improves the performance. Upon the generation of a packet, the node is required to wait until a new time slot starts before transmission is allowed. The proactive transmission of multiple copies of the same physical layer packet has been explored as a mean to improve the efficiency of the access policy. The diversity slotted ALOHA protocol [10] is shown to improve the relevant metrics such as packet loss rate (probability that a packet is not successfully received) and throughput (the average aggregate number of packets per packet duration successfully received), but unfortunately only for lightly loaded channels. When packet transmissions are sufficiently sporadic, sending multiple copies of the same data unit on the channel increases the probability that at least one is received successfully. On the other hand, when the average number of packets generated per unit of time increases beyond a threshold, replicating packets on the channel becomes detrimental due to the increase in multi-user interference. The breakthrough has been achieved when the replication of packets at the physical layer has been coupled with successive interference cancellation (SIC) at the receiver. A relevant example is the contention resolution diversity slotted ALOHA (CRDSA) protocol [11]: whenever a packet is successfully recovered, the receiver is able to retrieve the time instances of the transmitted packet copies and remove their interference of all transmissions. This operation may reduce the interference affecting other data units and possibly enable their recovery. The use of SIC opened a new research wave in the context of RA protocols and several extensions have been proposed in the recent past, that can be collected under the name of *modern random access* [12]. A number of enhancements for CRDSA have been proposed where the number of packet copies is drawn from a common, optimized, probability mass function [13,14,15]. The observation that SIC can be well modeled as a peeling process over a graph—as first observed in [13]—naturally enables the exploitation of tools borrowed from codes on graphs [16]. The throughput of a coordinated multiple-access scheme can be achieved under asymptotic assumptions (maximum number of packet copies and delay among them grows very large) for the destructive collision channel, as proved in [17]. The exploitation of SIC in RA continued well beyond CRDSA and included works such as [18,19]. The former merged the use of contention resolution tree algorithms [20] with interference cancellation, while the latter addressed the case in which the number of packet copies to be transmitted is not set a-priori but is dynamically adapted so as to reach a target throughput. Extensions that include interference cancellation for both ALOHA and spread spectrum ALOHA has been proposed in recent literature as well. In particular, contention resolution ALOHA (CRA) [21] proposed the use of replicas transmitted uniformly at random with no slot boundaries but within a MAC frame. Later, CRA has been extended by the use of selection- and maximal-ratio combining in [22]. In [23] the MAC frame synchronization is eliminated reducing the synchronization requirements on terminals. In [24], spread spectrum ALOHA is enhanced by the adoption of SIC at the receiver. Most of the modern random access protocols listed have been initially investigated assuming either the destructive collision channel model or a model for decoding based on a threshold. One of their main advantages is their analytically tractability, but it has to be mentioned that a real error correcting code has a non-negligible impact on the behavior of such schemes. In order to extend the evaluation beyond the destructive collision channel, the authors of [25,26,27] used a polynomial interpolation of the additive white gaussian noise (AWGN) channel code block error rate to determine when packets can be successfully decoded. This approach is exploited to analyze enhanced-spread spectrum ALOHA (E-SSA) in [25], CRDSA in [26] and irregular repetition slotted ALOHA (IRSA), coded slotted ALOHA (CSA) in [27].

The need for energy efficient uncoordinated multiple access solutions for IoT communications called for exploring information theoretic limits, and has been addressed in a recent work by Polyanskiy [28]. The finite-length achievability random coding bound to the *massive random access problem* [28]—later improved in [29]—opened the door to a new and flourishing wave of interest in RA solutions. Two main classes arise as promising novel attempts: the first concerns itself with compressed sensing-based solutions represented by works such as [30,31,32,33,34,35,36,37], while the second class relies on conventional channel code-based schemes characterized by works such as [38,39,40,41,42,43,44,45]. By no means we consider to have given an exhaustive listing of each class. The key aspect addressed in the aforementioned works is to reduce the decoding complexity. The authors in [30] propose an algorithm employing binary chirp coding amenable of low-complexity decoding. Vem and co-authors [31] solve the complexity issue by following a divide and conquer idea, further expanded on in [32] and in [34,35]. Exploring alternatives to reduce transmitter complexity, Truhachev and co-authors [33] propose a modification of [31] leveraging spreading techniques. In the second class of solutions, Ordentlich and Polyanskiy [38] propose the use of a time-slotted scheme and a receiver based on the concatenation of an inner binary code with an outer code. In the quest to improve the performance of the scheme in [38], a single-user Polar decoder augmented with SIC is proposed in [39]. Pradhan and co-authors [40] take an alternative path by adopting the use of random spreading and a polar code. In [43] they extend their work by enabling soft-input soft-output minimum means square error estimation. Zheng and co-authors, in [41], build upon [40] by using sparse spreading, where codewords are zero-padded and interleaved based on a portion of the message to be transmitted. Tanc and Duman [42] explore the use of convolutional coding together with random signatures. Han and co-authors in [44] also split the message into two parts and adopt sparse spreading with index modulation as one component code, while the other is protected with a tail biting convolutional code. Finally, Ahmadi and Duman in [45] propose an extension of [40] by letting users transmit with different power levels. An optimization problem is defined to identify the required number of power levels and their values.

### 1.1. Main Contributions and Structure of the Paper

Despite the several enhancements proposed in the recent past, ALOHA and SA, with minor variations, are still adopted in the current mobile communication standard for the log-on procedure [46] as well as in a number of IoT communication technologies as, for example, SigFox [47] and LoRaWAN [48]. The use of the destructive collision channel, i.e., collision free packets are retrieved with probability one, while packets involved in a collision are always regarded as lost, is a viable physical layer abstraction for SA, but its use in ALOHA becomes unconvincing. In fact, the presence of forward error correction (FEC), yields to partial protection against not only the effects of the channel but also against some level of multi-user interference. Since in ALOHA most of the collisions are only partial, regarding all of them as destructive may be implausible. Interestingly, to the authors’ knowledge, an analytical model able to capture the beneficial impact of FEC on the ALOHA protocol is still missing. Along this line of research we tried to close this gap and in particular we focused on the following aspects:

- We characterize the ALOHA protocol with an analytical model able to include the effect of FEC. We rely on a decoding condition based on a threshold which is function of the code rate and assume a capacity achieving channel code. Under the assumption of perfect power control, we are able to derive exact expressions for both the packet loss rate and the spectral efficiency;
- We explore a time- and frequency-asynchronous ALOHA scheme protected with FEC and extend the analytical analysis to this case as well. Packets are allowed to be sent not only whenever generated from higher layers, but are also randomized in frequency within a given channel bandwidth;
- We compare the two schemes, i.e., ALOHA and time and frequency ALOHA with FEC, shedding light on the still unexplored tradeoffs. While time and frequency ALOHA under the destructive collision channel is known to under perform compared to ALOHA [49,50], when packets are instead protected by FEC, the two schemes have a comparable performance;
- Finally, we consider a practical low-Earth orbit (LEO) communication system serving an IoT application and mimic the performance of SigFox compared to a similar system where packets are protected by FEC.

The remainder of this paper is structured as follows. After introducing the system model in Section 2, we provide in Section 3 the analytical analysis for both ALOHA (Section 3.1) and time and frequency ALOHA (Section 3.2), both aided by FEC. The numerical results are presented and discussed in Section 4, highlighting some fundamental tradeoffs and comparing the effectiveness of ALOHA and time and frequency ALOHA. Finally, Section 5 draws final remarks and offers some relevant open issues and future research directions.

### 1.2. Notation

We denote random variables by uppercase seriffed letters, while we refer to their realizations in lowercase, e.g., *X* and *x*. Vectors are denoted with bold lowercase letters, e.g., x. The expectation operator is denoted as E[·] and the real and imaginary part operators are represented with ℜ{·} and ℑ{·}, respectively. Units of measurements for variables and constants are introduced by the square brackets notation [·] and we use the short form *b* for referring to bits, *pk* for packets and *sym* for symbols.

## 2. System Model and Preliminaries

In our contribution we will focus on the return uplink of a satellite communication system serving low-cost low-power IoT nodes. In this regard, terminals are equipped with a single-antenna transmitter-only radio link. We assume that the devices access the common wireless medium following the ALOHA RA policy [51], i.e., nodes transmit over the channel in an uncoordinated fashion regardless of other users’ activity, and without retransmissions. No time-slot synchronization is required, reducing the terminal cost and battery consumption. Leaning on the typical assumption of infinite user population, the aggregate channel traffic is modeled by a Poisson distribution of parameter λ [b/s/Hz], or $\lambda^{\prime }=\lambda /k$ [pk/s/Hz], with *k* representing the number of information bits in a data unit.

In order to improve reliability of the transmissions and counteract in part the effect of the channel and multiple access interference, data units are protected with FEC characterized by a Gaussian codebook with code rate $R=k/n_{s}$ [b/sym], where $n_{s}$ is the number of symbols composing a packet. Let us define the packet duration $T_{p}$ and the symbol duration $T_{s}$, clearly $T_{p}=T_{s}n_{s}$. The *normalized channel load* G measured in [pk/pk duration and pk bandwidth] captures the average number of packets transmitted during one data unit duration and per transmission bandwidth W. The normalized channel load is related to λ and $\lambda^{\prime }$ by:

(1) $$G=\frac{\lambda }{R}=\lambda^{\prime }T_{p}W,$$

where we assume an ideal pulse shaping filter, i.e., $T_{s}=1/W$.

We consider transmissions over an AWGN channel. The discrete-time received the signal of the packet sent by the *u*-th user $y^{\left(u\right)}=\left[y_{1},\cdots ,y_{n_{s}}\right]$ can be written as:

$$y^{\left(u\right)}=x^{\left(u\right)}+z^{\left(u\right)}+n.$$

With $z^{\left(u\right)}$ being the aggregate multi-user interference over the considered codeword, and $n=\left[n_{1},\cdots ,n_{n_{s}}\right]$ being the noise vector whose elements are sampled from a zero-mean complex white Gaussian process with variance $\sigma^{2}$ per complex dimension, i.e., $n_{h}∼\mathrm{CN}\left(0,2\sigma^{2}\right)$ for $h=1,\cdots ,n_{s}$. We will consider ideal estimate of sampling epoch, frequency and phase offsets, hence $x^{\left(u\right)}=\left[c_{1}^{\left(u\right)},\cdots ,c_{n_{s}}^{\left(u\right)}\right]$ is the transmitted codeword of user *u*. Let us denote with $P_{h}^{\left(u\right)}=E\left[|c_{h}^{\left(u\right)}{|}^{2}\right]$ the received power of the useful signal for the *h*-th symbol, with $N=2\sigma^{2}$ the noise power and with $Z_{h}^{\left(u\right)}=E\left[|z_{h}^{\left(u\right)}{|}^{2}\right]$ the aggregate interference power on symbol *h*. Then, the signal-to-interference plus noise ratio γ, for the *h*-th received symbol of the user’s *u* data unit can be computed as:

(2) $$\gamma_{h}^{\left(u\right)}=\frac{P_{h}^{\left(u\right)}}{N+Z_{h}^{\left(u\right)}}.$$

By assuming perfect power control, i.e., all users are received with equal power, we can simplify $P_{h}^{\left(u\right)}=P$. Hence, Equation (2) reduces to:

$$\gamma_{h}^{\left(u\right)}=\frac{P}{N+Z_{h}^{\left(u\right)}}.$$

**Remark** **1.** *Very low-cost IoT terminals will hardly have the capability to adapt their transmission power so as to be received with the same power at the base station or satellite. Nonetheless, IoT will cover a very broad range of scenarios and for some of them terminals may have a more powerful hardware capable of, for example, adapt the transmission power. This is the case for SigFox and LoRa transmitters. In this latter scenario, two ways for providing perfect power control can be foreseen. For very static scenarios (fixed terminals, fixed receiver and static channel) open-loop static power assignment can be utilized. For non-static scenarios closed-loop may be preferred instead. This would be the case for an LEO communication system where providing the same power at the receiver input (the LEO satellite) entails a quite complex algorithm that shall measure the received power, for example via a beacon, and adapt the transmission power accordingly.*

We now define the two metrics we will consider throughout the contribution, i.e., packet loss rate (PLR) and spectral efficiency.

**Definition** **1** (Packet loss rate)**.** *The packet loss rate $p_{l}$ is the probability that a data unit is not correctly received.*

**Definition** **2** (Spectral efficiency)**.** *The spectral efficiency S is the average number of (information) bits correctly received per unit of time and frequency.*

### 2.1. Modelling the Decoding Process

In the classic literature on RA protocols, the destructive collision channel has been used as introductory yet very useful channel model abstraction [51]. Nonetheless, when packets are protected with forward error correction and asynchronous transmissions are allowed, assuming that a packet is lost, even if the vast majority is collision-free, is particularly pessimistic (see discussions in [22]). In the quest for more accurate abstractions of the physical layer that are able to capture the benefit of FEC, several attempts have been made in recent works, e.g., [21,22,23].

Assume that the packet *u* interfered with j∈N other packets with an overlap of $x_{h}\in \left(0,1\right]$ for h=1,⋯,j. By leaning on the approach in [21,52] we will compute the mean of the signal-to-interference and noise ratio (SINR) $\bar{\gamma }$ observed over the generic packet *u* as:

$${\bar{\gamma }}^{\left(u\right)}=\frac{P}{N+\left(\sum_{h=1}^{j}x_{h}^{\left(u\right)}\right)P}=\frac{P}{N+{\bar{Z}}^{\left(u\right)}}.$$

Choosing the code rate R, the data unit *u* is declared as correctly decoded if:

(3) $$R<\log_{2}\left(1+{\bar{\gamma }}^{\left(u\right)}\right).$$

By recalling the assumption of the Gaussian codebook (hence Gaussianity of the interference), the decoding condition assumes that messages are long enough so that the rate can approach the capacity of the AWGN channel.

Before entering in the details of the packet loss rate and spectral efficiency analysis, we would like to make few observations on the model for successful decoding presented in this Section.

### 2.2. Discussion on the Model for Successful Decoding

For the analysis of the performance of asynchronous RA protocols, a simple yet insightful abstraction of the physical layer able to capture the relevant tradeoffs has always been key. Few relevant alternatives beyond the selected model have been proposed in the recent past.

Targeting a similar level of precision, we can find the model based on instantaneous mutual information as presented in [22]. Resorting to a parallelism with the *block interference channel* [53], the authors consider the mutual information carried by each packet symbol, similarly to [54], and compute its average over the entire data unit. The result is compared with the chosen channel code rate and a decision of successful decoding is taken if the rate lies below the computed average mutual information. Similarly to the model chosen for our analysis, the PHY abstraction based on mutual information condenses the multi-dimensional problem of multi-user interference affecting each transmission, that may unfold in an untractable number of subcases, into a one-dimensional comparison of a function of the SINR with the selected code rate R. Compared to the model adopted in our work, the one leaning on mutual information provides more conservative results. In fact, it can be proven via Jensen’s inequality that the right hand side of Equation (3) is always greater than or equal to the right hand side of Equation (7) in [22].

Introducing a higher level of detail would, for example, entail the inclusion of the PLR performance of a code family or a specific channel code. The SINR experienced by each data unit can be used to inspect the PLR performance of the error correcting code, so as to make a decision about whether the packet can be correctly decoded, similar to what was proposed in [26,27,55] although for a time-slotted scenario, or as in [25] for spread spectrum ALOHA. Even though more precise than the adopted PHY abstraction, such approximation still holds similar limitations:

- the variability of the interference level along the packet is difficult to be captured. In a packet collision, how the multi-user interference hits the packet to be decoded has a fundamental role in determining whether it can be decoded or not. This is a new dimension beyond the total level of interference experienced. Unfortunately also here a function of the SINR is required to make use of the PLR, hence compressing this information in just one single value and losing the time dependance. Let us consider, for example, the simple case in which a packet is collided with a single other by say 50%. For the considered model this scenario is equivalent to one in which the packet is collided with two other packets, but only with 25% of its duration. This is because the interference scenario is mapped to a 1-dimensional number which is the average SINR over the packet. Clearly, depending on whether the 25% of each collision happens on the same portion of the packet or not, a real FEC code may behave drastically differently. In fact, if the 25% collided is on the same packet portion, this would result in fewer interfered symbols but with a lower SINR. The other case instead would result in more symbols interfered but with a higher SINR;
- interference is assumed to be Gaussian-like to enable the use of the PLR performance as relevant metric. The Gaussianity of interference is a good approximation when the number of interferers is not too small, or with few interferers whose signals bear imperfections as time-, frequency- and phase-offsets [55].

A further refinement of the decoding procedure would require the choice of an exact channel code together with the decoding algorithm adopted. In this way, full physical layer simulations may be required. This approach goes beyond the scope of this work, which is to provide a flexible and fast tool to investigate relevant tradeoffs for ALOHA with FEC.

## 3. Packet Loss Rate and Spectral Efficiency Analysis of ALOHA with Forward Error Correction

In this section we elaborate an analytical model able to predict the PLR and the spectral efficiency performance of ALOHA with forward error correction in two different flavors. We start in Section 3.1 by considering a scenario where the transmitted messages are long enough so that the rate can approach the capacity of the AWGN channel. In Section 3.2, taking inspiration by relevant technologies widely adopted in the IoT world like SigFox [47] and LoRaWAN [48] (especially in its latest evolution named LoRa-E [56]), we extend the analysis by considering an ALOHA multiple access scheme, where the transmission bandwidth is smaller than the channel bandwidth and users send their data units uniformly at random within the available channel. In this second scenario we also assume that the transmitted messages are long enough so that the rate can approach the capacity of the AWGN channel with a vanishingly small error probability.

### 3.1. Forward Error Correction ALOHA

We are now interested in characterizing the average PLR $p_{l}$ of an ALOHA multiple access protocol where packets are protected with a Gaussian codebook of rate R. Denote with *J* the r.v. representing the number of interferers affecting a transmission. We can compute the average PLR by conditioning on the number of interferers (J=j) and then removing the conditioning as:

(4) $$p_{l}=1-\sum_{j=0}^{\infty }\mathrm{Pr}\left(u\;\mathrm{successful}|J=j\right)\mathrm{Pr}\left(J=j\right).$$

Once the packet loss rate is available, i.e., when Equation (4) is solved, the spectral efficiency can be computed following:

$$S=G(1-p_{l}).$$

We denote with $p_{u|j}≜\mathrm{Pr}\left(u\;\mathrm{successful}|J=j\right)$. Consider the transmission of the data unit *u*, the probability of having J=j packets interfering with *u* is equivalent to the probability that *j* terminals transmitted over two packet durations. Recall that the aggregate traffic follows a Poisson distribution of intensity G packets per packet duration (see also definition in Equation (1)), hence Equation (4) can be reformulated as:

(5) $$p_{l}=1-\sum_{j=0}^{\infty }p_{u|j}\;\frac{{\left(2G\right)}^{j}e^{-2G}}{j!}.$$

Let us now investigate $p_{u|j}$, starting with the case j=1, i.e., the packet is interfered with one single other packet.

**Case** j=1

To compute $p_{u|1}$ we can exploit the successful decoding condition of Equation (3). The probability of user *u* being decoded given that it was involved in a collision with one other packet is thus:

$$p_{u|1}=\mathrm{Pr}\left(R<\log_{2}\left(1+{\bar{\gamma }}^{\left(u\right)}\right)|J=1\right).$$

We define the useful quantity *normalized average interference power* ${\hat{Z}}^{\left(u\right)}$ as:

(6) $${\hat{Z}}^{\left(u\right)}≜\frac{{\bar{Z}}^{\left(u\right)}}{P}=X_{1}^{\left(u\right)},$$

with $X_{1}^{\left(u\right)}$ being an r.v. capturing the amount of overlap between the interfering packet and the data unit of interest. Clearly, $X_{1}^{\left(u\right)}$ is uniformly distributed within the range $\left(0,1\right]$ and consequently also ${\hat{Z}}^{\left(u\right)}∼U\left(0,1\right)$. The probability $p_{u|1}$ then becomes:

$$p_{u|1}=\mathrm{Pr}\left(R<\log_{2}\left(1+{\bar{\gamma }}^{\left(u\right)}\right)|J=1\right)=\mathrm{Pr}\left({\hat{Z}}^{\left(u\right)}<\frac{1}{2^{R}-1}-\frac{N}{P}|J=1\right).$$

Let us denote with $F_{\hat{Z}}\left(\hat{z}\right)$ the cumulative distribution function (CDF) of the uniformly distributed r.v. ${\hat{Z}}^{\left(u\right)}$. Obviously $F_{\hat{Z}}\left(\hat{z}\right)=\hat{z}$. For ease of notation let us also define δ as:

$$\delta ≜\frac{1}{2^{R}-1}-\frac{N}{P}.$$

The parameter δ can be interpreted as the minimum interference such that a packet cannot be successfully decoded. We observe that depending on the chosen rate R and on the received signal-to-noise ratio (SNR) P/N, δ may be larger than 1. Nonetheless, the r.v. ${\hat{Z}}^{\left(u\right)}$ has a support in $\left(0,1\right]$, therefore we may rewrite $p_{u|1}$ as:

(7) $$p_{u|1}=\mathrm{Pr}\left({\hat{Z}}^{\left(u\right)}<\delta |J=1\right)=F_{\hat{Z}}\left(\min(\delta ,1)\right)=\min\left(\delta ,1\right),$$

where min(δ,1) takes care of confining the evaluation of $F_{\hat{Z}}\left(\hat{z}\right)$ in the support of ${\hat{Z}}^{\left(u\right)}$. Note that for the case j=1 the minimum operation is not required, but it will be fundamental in the generalization for j>1.

**Case** j>1

**Figure 1:**
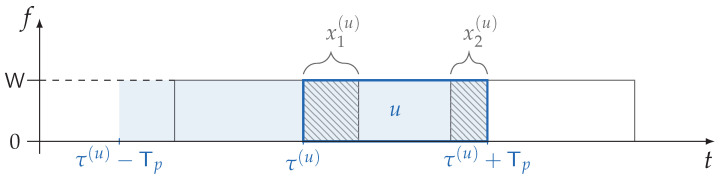
Example of a collision with two other packets, i.e., j=2. In this case we have $X_{1}^{\left(u\right)}=x_{1}^{\left(u\right)}$, $X_{2}^{\left(u\right)}=x_{2}^{\left(u\right)}$, and ${\hat{Z}}^{\left(u\right)}=x_{1}^{\left(u\right)}+x_{2}^{\left(u\right)}$. In general, the start of any packet in the range $\left(\tau_{0}-T_{p},\tau_{0}+T_{p}\right)$, represented by the shaded blue area, would cause a collision with the data unit of user *u*.

For reference, we show an example with j=2 interferers in Figure 1. When the number of interfering packets exceeds one, the normalized average interference power ${\hat{Z}}^{\left(u\right)}$ becomes the sum of *j* independent uniformly distributed r.v.s in $\left(0,\;1\right]$, i.e.,

(8) $${\hat{Z}}^{\left(u\right)}≜\frac{{\bar{Z}}^{\left(u\right)}}{P}=\sum_{h=1}^{j}X_{h}^{\left(u\right)}\;\mathrm{with}\;X_{h}^{\left(u\right)}∼U\left(0,1\right),\;\forall h=1,\ldots ,j.$$

The r.v. $X_{h}^{\left(u\right)}$ represents the amount of overlap the data unit *u* has with the *h*-th interfering packet. We may observe that ${\hat{Z}}^{\left(u\right)}$ follows the Irwin–Hall distribution, hence its CDF can be conveniently expressed as:

FZ^(z^;j)=1j!∑l=0⌊z^⌋(−1)ljl(z^−l)j.

Recalling that $F_{\hat{Z}}\left(\hat{z};j\right)$ is the CDF of the r.v. ${\hat{Z}}^{\left(u\right)}$, the probability that user *u* is correctly decoded when collided with *j* other packets is:

(9) pu|j=PrZ^(u)<δ|J=j=FZ^min(δ,j);j=1j!∑l=0⌊min(δ,j)⌋(−1)ljl(min(δ,j)−l)j.

**Remark** **2.** *Depending on the choice of the rate R and on the received SNR P/N, δ may be larger than j for some values of j∈N. The minimum operation min(δ,j) ensures that the CDF is computed in the support of ${\hat{Z}}^{\left(u\right)}$, i.e., $\left(0,j\right]$.*

**Remark** **3.** *The Irwin–Hall distribution includes as a special case the uniform distribution when j=1 and the triangular distribution for j=2. In fact, when j=1,*

FZ^(z^;1)=1j!∑l=0⌊z^⌋(−1)ljl(z^−l)jj=1=∑l=0⌊z^⌋(−1)l1l(z^−l)=z^,

*for $\hat{z}\in \left(0,1\right]$, which corresponds to the CDF of a standard uniform r.v.. In this way, Equation (9) includes Equation (7) as a special case for j=1.*

Thanks to the observation in Remark 2, we can finally compute the packet loss rate $p_{l}$ by substituting Equation (9) in Equation (5) as:

pl=1−∑j=0∞pu|j2Gje−2Gj!=1−∑j=0∞1j!∑l=0⌊min(δ,j)⌋(−1)ljl(min(δ,j)−l)j2Gje−2Gj!.

### 3.2. Forward Error Correction Time and Frequency ALOHA

When transmissions occupy a smaller bandwidth than the channel allocated for the system, the analysis provided in Section 3.1 shall be extended. In particular, we assume that transmissions are asynchronous both in time and frequency, i.e., when a transmission is triggered by the generation of a packet from higher layers, the central frequency is selected uniformly at random within the total channel bandwidth. This access method is especially relevant, as it is adopted by IoT technologies such as SigFox [47] and LoRaWAN (especially in LoRa-e) [48].

Let us define the channel bandwidth as B. We assume that it is much larger than the bandwidth occupied by the transmission of a single packet, i.e., B≫W, so that border effects can be neglected. Packets close to the limits of the channel bandwidth are subject to a lower level of interference simply because concurrent transmissions are allowed only within the channel band. For example, when packets are transmitted on the minimum frequency within the channel, interference can come from packets at the same frequency or higher, effectively reducing by a factor of two the probability of interference. One could take into considerations this effect following a similar path addressed in [50] and extend the analysis provided in the following.

As in the previous Section, we are interested in characterizing the average PLR $p_{l}$ of an ALOHA multiple access protocol where packets are protected with a Gaussian codebook of rate R. Following a similar path, we can compute the average PLR by conditioning on the number of interferers (J=j) and then removing the conditioning as:

(10) $$p_{l}=1-\sum_{j=0}^{\infty }p_{u|j}\mathrm{Pr}\left(J=j\right).$$

With respect to the previous scenario, both $p_{u|j}$ and Pr(J=j) have to be re-elaborated taking into account the modified access method. Furthermore, as we compute the packet loss rate by solving Equation (10), the spectral efficiency can be easily derived as follows:

$$S=G(1-p_{l}).$$

Following similar steps discussed in Section 3.1, we define $X_{h}^{\left(u\right)}$ as the r.v. representing the amount of interference caused by the *h*-th interferer on data unit *u*. Differently from the plain ALOHA scenario, the collision may partially happen both in the time and frequency domains. Therefore, $X_{h}^{\left(u\right)}$ captures the area of the collision between the two data units and hence is modeled as the product of two standard uniform r.v.s *U* and *V* (as we consider the power normalization as per Equations (6) and (8)). In this way, we have:

$$X_{h}^{\left(u\right)}=U_{h}^{\left(u\right)}V_{h}^{\left(u\right)}\;U_{h}^{\left(u\right)}∼U\left(0,1\right),\;V_{h}^{\left(u\right)}∼U\left(0,1\right).$$

For ease of notation we drop the subscript *h* and the superscript (u). We can compute the CDF $F_{X}\left(x\right)$ of *X* as:

$$F_{X}\left(x\right)=\mathrm{Pr}\left(X\leq x\right)=\int_{0}^{1}\mathrm{Pr}\left(V\leq \frac{x}{u}\right)f_{U}\left(u\right)du=\int_{0}^{x}du+\int_{x}^{1}\frac{x}{u}du=x-x\log_{e}\left(x\right).$$

As a consequence, the probability density function (PDF) $f_{X}\left(x\right)$ of *X* becomes:

(11) $$f_{X}\left(x\right)=-\log_{e}\left(x\right).$$

Conditioned on J=j interferers, the normalized average interference power follows ${\hat{Z}}^{\left(u\right)}=\sum_{h=1}^{j}X_{h}^{\left(u\right)}$, similarly to Equation (8), but with the fundamental difference that here the i.i.d. r.v.s $X_{h}^{\left(u\right)}$ are governed by the PDF in Equation (11). Such important difference is the direct consequence of the modified access scheme, now asynchronous both in time and frequency.

We are interested in computing the CDF of ${\hat{Z}}^{\left(u\right)}$ and we lean on the property of characteristic functions. In particular, since ${\hat{Z}}^{\left(u\right)}$ is the sum of *j* i.i.d. random variables $X_{h}^{\left(u\right)}$, its characteristic function $\varphi_{\hat{Z}}\left(t\right)$ can be written as the product of the characteristic functions $\varphi_{X_{h}^{\left(u\right)}}\left(t\right)$ of $X_{h}^{\left(u\right)}$. By applying the definition,

(12) $$\varphi_{X_{h}^{\left(u\right)}}\left(t\right)=E\left[e^{iX_{h}^{\left(u\right)}t}\right]=-\int_{0}^{1}e^{itx}\log_{e}\left(x\right)dx=\frac{i(-Ci\left(t\right)-iSi\left(t\right)+\log_{e}\left(t\right)+\beta )}{t},$$

with $Ci\left(t\right)=-\int_{t}^{\infty }\frac{\cos(\omega )}{\omega }d\omega$ being the cosine integral, $Si\left(t\right)=\int_{t}^{\infty }\frac{\sin(\omega )}{\omega }d\omega$ being the sine integral and $\beta =\int_{1}^{\infty }\left(-\frac{1}{\omega }+\frac{1}{\left\lfloor \omega \right\rfloor }\right)d\omega$ being the Euler–Mascheroni constant. Exploiting Equation (12), the characteristic function of ${\hat{Z}}^{\left(u\right)}$ can be easily derived as:

(13) $$\varphi_{\hat{Z}}\left(t\right)=\prod_{h=1}^{j}\varphi_{X_{h}^{\left(u\right)}}\left(t\right)={\left[\frac{i(-Ci\left(t\right)-iSi\left(t\right)+\log_{e}\left(t\right)+\beta )}{t}\right]}^{j}.$$

Thanks to the Gil–Pelaez theorem, the CDF $F_{\hat{Z}}\left(\hat{z};j\right)$ can be derived by the evaluation of the integral,

$$\begin{array}{cc} F_{\hat{Z}}\left(\hat{z};j\right) & =\frac{1}{2}-\frac{1}{\pi }\int_{0}^{\infty }ℑ\left\{\frac{e^{-it\hat{z}}\varphi_{\hat{Z}}\left(t\right)}{t}\right\}dt\\ & =\frac{1}{2}-\frac{1}{\pi }\int_{0}^{\infty }ℑ\left\{\frac{e^{-it\hat{z}}}{t}{\left[\frac{i(-Ci\left(t\right)-iSi\left(t\right)+\log_{e}\left(t\right)+\beta )}{t}\right]}^{j}\right\}dt, \end{array}$$

as a function of the characteristic function $\varphi_{\hat{Z}}\left(t\right)$ defined in (13).

Recalling that the data unit *u* is protected by a Gaussian channel code of rate R, *u* can be successfully received if and only if ${\hat{Z}}^{\left(u\right)}<\delta$, i.e., the total of interference (and therefore the overall overlap among interfering packets with *u*) does not exceed δ. In particular, the probability that user *u* is correctly decoded when colliding with *j* other packets is:

(14) $$\begin{array}{cc} p_{u|j} & =\mathrm{Pr}\left({\hat{Z}}^{\left(u\right)}<\delta |J=j\right)=F_{\hat{Z}}\left(\min(\delta ,j);j\right)\\ & =\frac{1}{2}-\frac{1}{\pi }\int_{0}^{\infty }ℑ\left\{\frac{e^{-it\left[\min(\delta ,j)\right]}}{t}{\left[\frac{i(-Ci\left(t\right)-iSi\left(t\right)+\log_{e}\left(t\right)+\beta )}{t}\right]}^{j}\right\}dt. \end{array}$$

**Figure 2:**
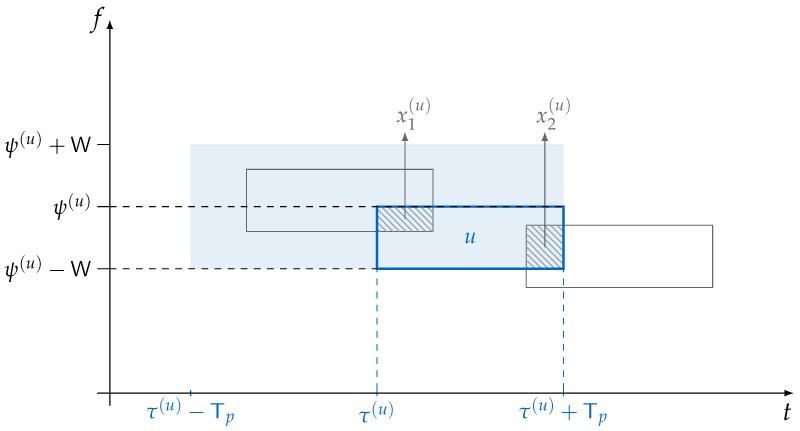
Time and frequency ALOHA, example of a collision with two other packets, i.e., j=2. In this case we have $X_{1}^{\left(u\right)}=x_{1}^{\left(u\right)}$, $X_{2}^{\left(u\right)}=x_{2}^{\left(u\right)}$, and ${\hat{Z}}^{\left(u\right)}=x_{1}^{\left(u\right)}+x_{2}^{\left(u\right)}$. In general, the start of any packet in the range $\left(\tau^{\left(u\right)}-T_{p},\tau^{\left(u\right)}+T_{p}\right)$ and $\left(\psi^{\left(u\right)}-W,\psi^{\left(u\right)}+W\right)$, represented by the shaded blue area, would cause a collision with the data unit of user *u*.

Observing the transmission of the generic packet *u* between $\tau^{\left(u\right)}$ and $\tau^{\left(u\right)}+T_{p}$ and in the band $\psi^{\left(u\right)}$, $\psi^{\left(u\right)}-W$ as depicted in Figure 2, the probability that J=j interfering packets clash with the data unit of interest is equivalent to the probability that *j* packets are transmitted in the area $4T_{p}W$ (shaded blue area in Figure 2). Recalling that the aggregate traffic is modeled following a Poisson distribution of intensity $\lambda^{\prime }$ [pk/s/Hz] we can write:

(15) $$\mathrm{Pr}\left(J=j\right)=\frac{{\left(4\lambda^{\prime }T_{p}W\right)}^{j}e^{-(4\lambda^{\prime }T_{p}W)}}{j!}=\frac{{\left(4G\right)}^{j}e^{-(4G)}}{j!},$$

by leaning on the definition of G in Equation (1).

Finally, substituting Equations (14) and (15) in Equation (10) we get:

$$\begin{array}{cc} p_{l} & =1-\sum_{j=0}^{\infty }\left[\left(\frac{1}{2}-\frac{1}{\pi }\int_{0}^{\infty }ℑ\left\{\frac{e^{-it\left[\min(\delta ,j)\right]}}{t}{\left[\frac{i(-Ci\left(t\right)-iSi\left(t\right)+\log_{e}\left(t\right)+\beta )}{t}\right]}^{j}\right\}dt\right)\right.\\ & \cdot \left.\frac{{\left(4G\right)}^{j}e^{-(4G)}}{j!}\right]. \end{array}$$

## 4. Numerical Results

In this Section we discuss numerical results of practical interest leaning on the derived analytical expressions of the packet loss rate and spectral efficiency found in Section 3.1 and Section 3.2.

**Figure 3:**
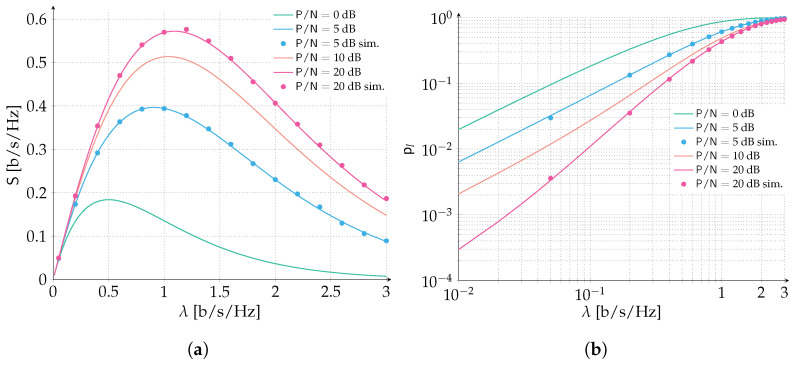
Performance of ALOHA with forward error correction. Data units are protected with a Gaussian codebook of rate R=1 bits per symbol. We select the SNR in the range $P/N\in \left\{0,\;5,\;10,\;20\right\}$ dB. Analytical results (continuous lines) are compared with Monte Carlo simulations (dots). (**a**) Spectral efficiency; (**b**) Packet loss rate.

We start by considering an ALOHA scenario where packets are protected with a Gaussian codebook of rate R=1 bits per symbol. Recall that packets are adopting perfect power control, and we let the P/N vary in the range $\left\{0,\;5,\;10,\;20\right\}$ dB. In Figure 3, we report the spectral efficiency (Figure 3a) and the packet loss rate (Figure 3b) for the aforementioned setting. When P/N=0 dB, δ=1−1=0. Recall that δ is the maximum amount of normalized interference that can be counteracted by the error correcting code still enabling the correct reception of the data unit. Hence, when δ=0 we are under the destructive collision assumption: every collision will cause a packet to be lost no matter how small the overlap. Inspecting both the spectral efficiency and packet loss rate, we can observe how the model is properly capturing this special case and reflects the throughput and packet loss rate of a classic ALOHA protocol. As one can expect, when we increase P/N beyond 0 dB the error correcting code is able to counteract an increasing level of interference which translates in an improved performance of the packet loss rate and spectral efficiency. Nonetheless, the highest performance gain is reaped by the increase of SNR from 0 dB to 5 dB, where the peak spectral efficiency is more than doubled (0.396 [b/s/Hz] vs. 0.184 [b/s/Hz]) and a target PLR of $10^{-1}$ is achieved for almost three times the channel load (0.16 [b/s/Hz] vs. 0.06 [b/s/Hz]). Instead, a more stringent PLR of $10^{-2}$ is achieved for 0.02 [b/s/Hz] vs. <0.01 [b/s/Hz], more than a two-fold improvement. Comparing the P/N scenario of 5 dB with 10 dB, the performance gain is more limited, and is reduced even more when confronting the 10 dB with the 20 dB. In order to show how the analytical prediction is exact, we validated two performance curves, 5 dB and 20 dB, with Monte Carlo simulations, that adopt the same model for the successful decoding of packets as assumed by the analysis.

**Figure 4:**
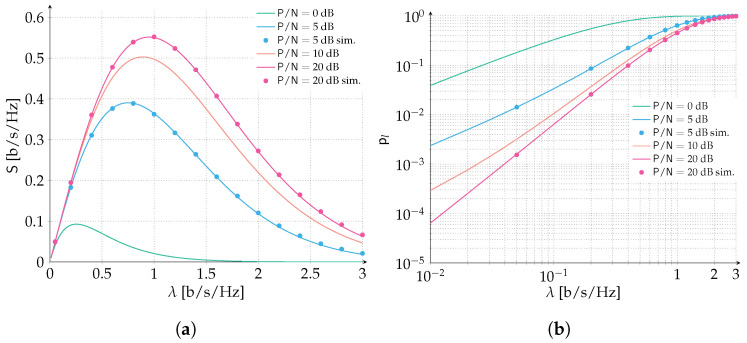
Performance of time and frequency ALOHA with forward error correction. The channel bandwidth to transmission bandwidth ratio is B/W=500. Data units are protected with a Gaussian codebook of rate R=1 bits per symbol. We select the SNR in the range $P/N\in \left\{0,\;5,\;10,\;20\right\}$ dB. Analytical results (continuous lines) are compared with Monte Carlo simulations (dots). (**a**) Spectral efficiency; (**b**) Packet loss rate.

In the second set of results, time and frequency ALOHA is investigated. Results are reported in Figure 4 for both spectral efficiency and packet loss rate (Figure 4a,b, respectively). Similarly to the ALOHA scenario, packets are protected with a Gaussian codebook of rate R=1 bits per symbol and we let the P/N vary in the range $\left\{0,\;5,\;10,\;20\right\}$ dB. Additionally, the channel bandwidth to transmission bandwidth is selected to be B/W=500 so that border effects can be neglected. As in the previous case P/N=0 dB corresponds to the destructive collision channel setup. Increasing the SNR allows the error correcting code to counteract some level of interference benefitting both packet loss rate and spectral efficiency. A very remarkable performance improvement can be already observed when considering the scenario P/N=5 dB. More than a four-fold increase in the peak spectral efficiency is observed (0.390 [b/s/Hz] vs. 0.093 [b/s/Hz]) and a target PLR of $10^{-1}$ is achieved for almost eight times the channel load (0.23 [b/s/Hz] vs. 0.03 [b/s/Hz]). Instead, a more stringent PLR of $10^{-2}$ is achieved for 0.04 [b/s/Hz] vs. ≪0.01 [b/s/Hz], more than a four-fold improvement. The outstanding performance improvement can be ascribed to the effect of forward error correction coupled with the access policy. In fact, when transmissions are asynchronous both in time and frequency, the probability that two or more packets collide increases with respect to the case in which transmissions occupy fully the channel bandwidth, under an equivalent channel load λ. On the other hand, the average overlap per packet collision is reduced by half. On average, a packet collision may involve half of the time duration and half of the frequency duration of a packet when time and frequency ALOHA is considered. These two effects partially compensate each other and when forward error correction is introduced, the time and frequency ALOHA policy largely benefits.

**Figure 5:**
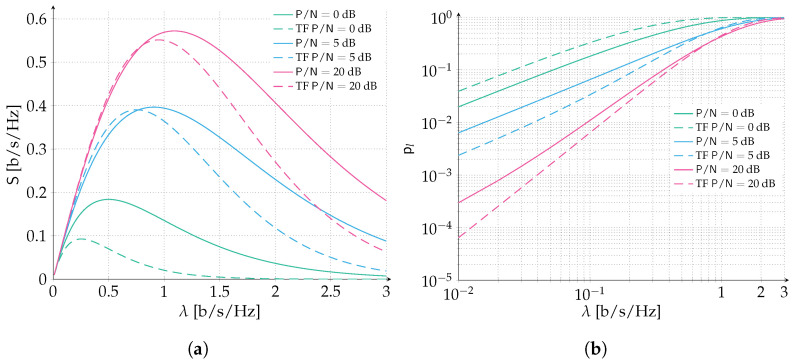
Performance of time and frequency ALOHA (dashed lines) compared with ALOHA (solid lines) both aided by forward error correction. The channel bandwidth to transmission bandwidth ratio is B/W=500 for time frequency ALOHA. Data units are protected with a Gaussian codebook of rate R=1 bits per symbol. We select the SNR in the range $P/N\in \left\{0,\;5,\;20\right\}$ dB. (**a**) Spectral efficiency. (**b**) Packet loss rate.

In order to compare the behavior of time and frequency ALOHA with ALOHA both aided by forward error correction we collect a subset of the previous results in Figure 5. The configuration is as before—packets are protected with a Gaussian codebook of rate R=1 bits per symbol and we let the P/N vary in the range $\left\{0,\;5,\;20\right\}$ dB. Additionally, the channel bandwidth to transmission bandwidth is selected to be B/W=500 so that border effects can be neglected. As expected, when the destructive collision channel scenario is considered, i.e., P/N=0 dB, ALOHA is able to largely outperform time and frequency ALOHA. However, when the presence of FEC can partially counteract the multi-access interference, the performance of time and frequency ALOHA are enhanced to competitive levels compared to ALOHA. Not only the peak spectral efficiency is now comparable for both P/N=5 dB and P/N=20 dB, but also for low to moderate channel loads the packet loss rate is outperforming the ALOHA policy. For example, a target PLR of $10^{-2}$ is achieved for two times the channel load (0.04 [b/s/Hz] vs. 0.02 [b/s/Hz]) in time frequency ALOHA w.r.t. to ALOHA for P/N=5 dB and the target PLR of $10^{-1}$ is achieved for a ∼44% larger channel load (0.23 [b/s/Hz] vs. 0.16 [b/s/Hz]). Similarly at P/N=20 dB, a target PLR of $10^{-2}$ is achieved for a ∼30% larger channel load (0.13 [b/s/Hz] vs. 0.10 [b/s/Hz]) and the target PLR of $10^{-1}$ is achieved for a ∼11% larger channel load (0.41 [b/s/Hz] vs. 0.37 [b/s/Hz]). The remarkable performance improvement can be identified in two counterbalancing effects. Fixing the channel load, the probability that *k* users collide is larger for time and frequency ALOHA compared to ALOHA. On the other hand for a fixed number of interferers the probability that the level of interference is smaller or equal than a certain value is always larger in the former case. Hence, these two effects are influencing the performance in two opposite directions, the first is worsening the packet loss rate (higher number of interferers) while the second is improving it (lower level of interference). As a remark, it is important to stress that an ALOHA system operated in time and frequency aided with forward error correction provides a better performance for all channel load values of practical interest, as we observed by pointing to possible target PLR with respect to an ALOHA policy. This is in contrast with what is known from existing literature when the destructive collision channel model is considered. The protecting effect of channel coding also against multi-user interference is indeed particularly beneficial.

**Figure 6:**
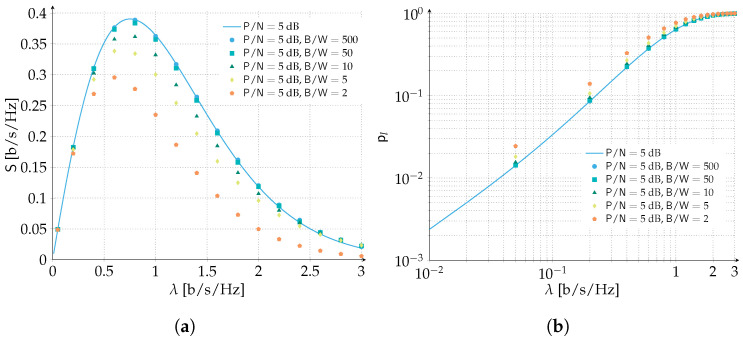
Performance of time and frequency ALOHA aided by forward error correction for various channel bandwidth to transmission bandwidth ratios. The channel bandwidth to transmission bandwidth ratio is in the range B/W={500,50,10,5,2}. Data units are protected with a Gaussian codebook of rate R=1 bits per symbol. We select the SNR of P/N=5 dB. Similar trends have been observed also for other values of SNR not reported in the figure. (**a**) Spectral efficiency. (**b**) Packet loss rate.

The analytical analysis of time and frequency ALOHA assumes that the channel bandwidth to transmission bandwidth ratio is large enough so that border effects can be neglected, i.e., B/W→∞. We already observed that for B/W=500 such assumption holds true and numerical results are perfectly predicted by the analysis, as observed in Figure 5. To answer the practical question on what is the smallest value of the ration B/W for which border effects can be neglected, we performed extensive numerical simulations and the results are reported in Figure 6 for both the packet loss rate and the spectral efficiency. While reducing the B/W down to 50 has a very limited impact on the performance, and hence can be still very well predicted with the presented analytical model, a further reduction to 10 shows the first visible differences. Below B/W=10 the spectral efficiency reduction becomes particularly visible and the analytical model starts to become loose w.r.t. the numerical results. The peak spectral efficiency is reduced from 0.39 [b/s/Hz] to 0.3 [b/s/Hz] when B/W=2 and the spectral efficiency is largely overestimated for all channel load values beyond the peak spectral efficiency. Similarly, the PLR is visibly worse than the predicted analytical performance for all channel load values. Hence, below B/W=50, the analytical model shall be extended to take into account the border effects, if a better performance estimate is required. Similar arguments as presented in [50] can be exploited to include this effect in the packet loss rate analysis.

**Figure 7:**
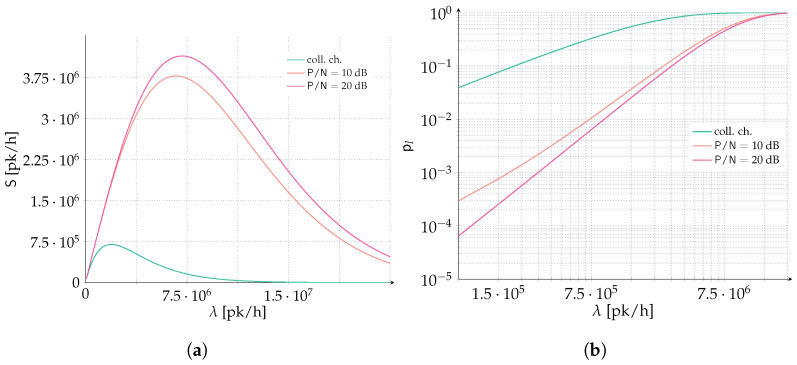
We consider a system similar to SigFox and we compare uncoded transmissions with data protected with forward error correction. We assume the return uplink of an LEO satellite communication system in the 860 MHz ISM band, with a satellite flying at 575 km, a maximum EIRP of 16 dBmW and a G/T of −22 dBi/K. An SNR between 18 and 22 dB could be achieve at the satellite for terminals at $40^{{}^{\circ}}$ and $90^{{}^{\circ}}$ elevation angle respectively. Hence, to be conservative we present results for P/N=10 dB and P/N=20 dB and for the uncoded case. The transmission band is W=100 Hz, and packets are sent asynchronous both in time and frequency. The total available band (channel bandwidth) is B=200 kHz resulting in a B/W=2000. The packet duration coincides with the maximum available payload of SigFox, i.e., 12 bytes or k=96 bits. We compare this uncoded scenario with a system employing forward error correction with rate R=1, emulating the possible choice of QPSK modulation coupled with a code rate 1/2 channel code. (**a**) Spectral efficiency. (**b**) Packet loss rate.

Finally, we report in Figure 7 the possible performance improvement of a time and frequency ALOHA system similar to SigFox when forward error correction is enabled. In SigFox, packets are transmitted uncoded over a very narrow transmission band of only W=100 Hz, asynchronous both in time and frequency. The total available band (channel bandwidth) is instead B=200 kHz resulting in a B/W=2000. We choose as packet duration the maximum available payload of 12 bytes, i.e., k=96 bits. We compare this uncoded scenario with a system employing forward error correction with rate R=1 [b/sym], emulating the possible choice of quadrature phase shift keying (QPSK) modulation coupled with a channel code rate 1/2 (recall that the rate R used in the entire manuscript always considers the effect of modulation and channel code combined, as it is measured in bits per symbol). We assume the return uplink of a LEO satellite communication system in the 860 MHz industrial scientifical and medical (ISM) band, with a satellite flying at 575 km, a maximum effecive isotropic radiated power (EIRP) of 16 dBmW and a G/T of −22 dBi/K. An SNR between 18 and 22 dB could be achieved at the satellite for terminals at $40^{{}^{\circ}}$ and $90^{{}^{\circ}}$ elevation angle respectively. We consider a simple clear sky AWGN channel with no additional losses (antenna losses, atmospheric losses, terminal losses etc. are all considered to be negligible). Finally, no geometry of the link is taken into account, i.e., perfect power control is considered so that the received power at the satellite antenna input is the same for every transmission. Clearly this is a simplified satellite setting, nonetheless it can shed light on the possible gain one could expect if forward error correction is adopted. Analytical results are collected in Figure 7 for both the spectral efficiency, Figure 7a and packet loss rate, Figure 7b. Differently from previous results, here we are concerned with the number of packets per hour, denoted with [pk/h] in the figures, that can be served by the system. This measure is more suited for system design, since it can be easily adapted to the IoT use case at hand. Typically, an IoT system is designed assuming terminals reporting data a given amount of times per day. Three set of curves are presented, two consider R=1 and P/N=10 dB and P/N=20 dB, while the last curve (green and denoted by *coll. ch.* in the plots) is for an uncoded system. Looking at Figure 7a for an SNR of 10 dB, a five fold increase in the peak of spectral efficiency can be achieved compared to an uncoded system, reaching up to $3.75\cdot 10^{6}$ [pk/h]. Even more, for a target packet loss rate of $10^{-1}$ more than ten times the aggregate channel traffic can be supported when forward error correction is present compared to the uncoded system. Finally, for a more stringent PLR constraint of $10^{-2}$ up to $7.5\cdot 10^{5}$ [pk/h] and $10^{6}$ [pk/h] can be supported for P/N=10 dB and P/N=20 dB, respectively.

## 5. Conclusions and Outlook

We have studied the effect of forward error correction (FEC) on the performance of two random access systems: ALOHA, where the channel bandwidth is confined to the transmission bandwidth, and ALOHA in time and frequency, where the channel bandwidth is instead much larger. A mathematical model that can accurately predict the packet loss rate and the spectral efficiency of said systems has been developed and validated via Monte Carlo simulations. The analysis yielded valuable and yet unexplored insights. In particular, we have observed that the use of FEC can be more beneficial to a time and frequency ALOHA policy rather than ALOHA. When the destructive collision channel is concerned, ALOHA is able to largely outperform time and frequency ALOHA. Instead, when FEC is adopted, comparable results in terms of both packet loss rate and spectral efficiency can be achieved. Furthermore, bandwidth border effects have been studied under various channel-to-transmission bandwidth ratios. Our model accurately predicts packet loss rates and spectral efficiencies for ratios greater than 50, which are typically to be found in real systems. Finally, we have shown that SigFox-like systems targeting the return uplink of satellite communication systems can greatly benefit from the use of FEC, thus achieving a multi-fold increase of the spectral efficiency for practical operational settings.

The present contribution aims at stimulating relevant work along this line of research. Several relevant open questions remain, for example, the extension of the analysis to fading channels, or to the use of optimized transmission power unbalance among terminals, which is expected to leverage the capture effect. Additionally, there is the possibility to include the border effects in the analysis so as to be able to cope with small B/W, or to analyze other relevant access policies such as diversity ALOHA with FEC.

## Data Availability

Not applicable.
